# Analysis of gut bacteriome of *in utero* arsenic-exposed mice using 16S rRNA-based metagenomic approach

**DOI:** 10.3389/fmicb.2023.1147505

**Published:** 2023-09-29

**Authors:** Shagun Shukla, Ankita Srivastava, Digvijay Verma, Siddhartha Gangopadhyay, Anchal Chauhan, Vikas Srivastava, Savita Budhwar, Dushyant Tyagi, Deepak Chand Sharma

**Affiliations:** ^1^Department of Microbiology, Dr. Shakuntala Misra National Rehabilitation University, Lucknow, Uttar Pradesh, India; ^2^Systems Toxicology and Health Risk Assessment Group, Council of Scientific & Industrial Research-Indian Institute of Toxicology Research (CSIR-IITR), Lucknow, Uttar Pradesh, India; ^3^Department of Environmental Microbiology, School of Earth and Environmental Sciences, Babasaheb Bhimrao Ambedkar University, Lucknow, India; ^4^Academy of Scientific Innovation and Research (AcSIR), Ghaziabad, India; ^5^Department of Nutrition Biology, Central University of Haryana, Mahendragarh, Haryana, India; ^6^Department of Mathematics and Statistics, Dr. Shakuntala Misra National Rehabilitation University, Lucknow, Uttar Pradesh, India

**Keywords:** *in-utero* arsenic exposure, bacteriome, arsenic, 16S rRNA gene, insulin signaling

## Abstract

**Introduction:**

Approximately 200 million people worldwide are affected by arsenic toxicity emanating from the consumption of drinking water containing inorganic arsenic above the prescribed maximum contaminant level. The current investigation deals with the role of prenatal arsenic exposure in modulating the gut microbial community and functional pathways of the host.

**Method:**

16S rRNA-based next-generation sequencing was carried out to understand the effects of *in utero* 0.04 mg/kg (LD) and 0.4 mg/kg (HD) of arsenic exposure. This was carried out from gestational day 15 (GD-15) until the birth of pups to understand the alterations in bacterial diversity.

**Results:**

The study focused on gestational exposure to arsenic and the altered gut microbial community at phyla and genus levels, along with diversity indices. A significant decrease in firmicutes was observed in the gut microbiome of mice treated with arsenic. Functional analysis revealed that a shift in genes involved in crucial pathways such as insulin signaling and non-alcoholic fatty liver disease pathways may lead to metabolic diseases in the host.

**Discussion:**

The present investigation may hypothesize that *in utero* arsenic exposure can perturb the gut bacterial composition significantly as well as the functional pathways of the gestationally treated pups. This research paves the way to further investigate the probable mechanistic insights in the field of maternal exposure environments, which may play a key role in epigenetic modulations in developing various disease endpoints in the progeny.

## Introduction

Arsenic is a common toxic contaminant found in the environment and is categorized as a group I carcinogen (Bhattacharya et al., 2007; IARC Working Group on the Evaluation of Carcinogenic Risks to Humans, 2012). Many populations around the world are exposed to above-permissible levels of arsenic via drinking water [World Health Organization and the U.S. Environmental Protection Agency (EPA)]. The established guidelines for arsenic are <10 μg/L (Hughes et al., 2011). Various epidemiological and animal studies have linked chronic arsenic exposure with cancers of various organs such as the skin, bladder, and liver, along with non-cancer endpoints such as diabetes, obesity, hypertension, and cardiovascular diseases (van de Wiele et al., 2010; Hughes et al., 2011; Gribble et al., 2012; Naujokas et al., 2013; Grau-Perez et al., 2018). Recently, many studies have shown the correlation between arsenic exposure and metabolic disorders (Paul et al., 2011; Maull et al., 2012; Castriota et al., 2018). Trillions of microbes are present in the human gut and work in symbiosis with the host to maintain vital functions such as food digestion, metabolic processing, immune system development, epithelial homeostasis, and xenobiotic biotransformation (Ley et al., 2006a,b; Young et al., 2008; Qin et al., 2010; Nicholson et al., 2012; Jandhyala et al., 2015). In recent years, many reports have indicated a link between perturbed gut microbiota and the cause of many diseases such as cancer, diabetes, cardiovascular diseases, and obesity (Ley et al., 2005, 2006b; Wang et al., 2011; Qin et al., 2012; Roderburg and Luedde, 2014; Gentile and Weir, 2018; Guglielmi, 2018). Besides, the composition of gut microbiota is known to be affected by a variety of external factors such as antibiotics, food, stress, drugs, and environmental chemicals such as arsenic, mercury, and hydrocarbons (Liebert et al., 1997; van de Wiele et al., 2005, 2010; Pinyayev et al., 2011; Choi et al., 2013; Zhang et al., 2015a,b; An et al., 2019). Linking altogether, it can be hypothesized that arsenic has the potential to perturb the composition of the gut microbiota and thus alter the metabolic state of the organisms.

Some findings are able to establish the relationship between arsenic exposure, gut microbiota, and metabolic disorders in the adult population (Lu et al., 2014; Hur and Lee, 2015; Brabec et al., 2020; Yang et al., 2021). The gut microbiota of the infant is primarily established during birth and early life and is transferred vertically from the mother (Yao et al., 2021). Alteration of the gut microbiota and metabolic disorders due to prenatal arsenic exposure has not yet been well-studied. Therefore, the present investigation planned to study the effect of prenatal arsenic exposure on the gut microbiota and metabolic profile of resident microbes.

## Materials and methods

### Animal treatment

Female Balb/C mice (6–8 weeks old) weighing between 25 and 28 g were acquired from the animal housing and rearing facility of CSIR-IITR, Lucknow, India. Animals were kept in polypropylene cages at 25°C with 12 h light/12 h dark cycles and had *ad libitum* access to purified water and a standard chow diet. The female mice were divided into three groups (*n* = 10 in each group), viz., (i) control (without arsenic exposure), (ii) 0.04 mg/kg as exposure group (low dose LD), and (iii) 0.4 mg/kg as arsenic group (high dose HD). A working dose of arsenic, i.e., 0.04 and 0.4 mg/kg, was freshly prepared by dissolving sodium (meta) arsenite (NaAsO_2_) in water and was given daily via oral gavage from GD-15 (gestational day minus 15; i.e., 15 days prior to mating) until delivery (i.e., GD-21; Sharma et al., 2022). Water was administered orally to the control group. Animals were set for mating in a 2:1 (female:male) ratio after 15 days of dosing and on getting a positive plug; the males were separated. The pups were housed with their mothers for weaning until 21 days after birth. After weaning, the male and female offspring were segregated, and three male pups from each group were sacrificed at 6 weeks. For studying the bacterial diversity, gut microbiota samples were procured by collecting fecal samples from the respective groups. The fecal collection was carried out under aseptic conditions using a sterile spatula. All the experimental procedures performed in this study were approved by the Institutional Animal Ethical Committee (IAEC) of the Council of Scientific and Industrial Research—Indian Institute of Toxicology Research (CSIR-IITR), Lucknow, India.

### Sample collection, DNA extraction, and sequencing

Fecal samples of 6-week-old mouse offspring were collected via standard protocol, and their metagenomic DNA was extracted using a QIAamp Power Fecal Pro DNA Kit—QIAGEN (Germany) and stored at −20°C until use. The extracted metagenomes were processed for amplification of the bacterial-specific 16S rRNA V_3_-V_4_ hypervariable region. Amplicon library was prepared by Qubit quantification of 40 ng of extracted fecal metagenome for amplifying the V_1 − 3F_ (5′AGAGTTTGATGMTGGCTCAG3′) and V (5′TTACCGCGGCMGCSGGCAC3′) hypervariable regions of the 16S rRNA encoding gene using the TAQ Master MIX (Biokart India Pvt. Ltd., India). Furthermore, next-generation sequencing (NGS) was performed using the Illumina Miseq platform with a 2 × 300 paired-end V_3_ sequencing kit at Biokart India Pvt. Ltd., Bangalore, Karnataka, India.

### Denoising and taxonomy assignment

Raw reads obtained from the Illumina sequencing were further denoised using DADA2 in QIIME2 (2020.11) for quality filtering, removal of chimeric sequences, and generation of Amplicon Sequence Variants (ASVs; Bolyen et al., 2019). Further good-quality reads obtained after denoising were processed for taxonomy assignment in QIIME2 (2020.11) by training a feature classifier in the SILVA 16S database to obtain various taxa (phylum, class, order, family, and genus) level distributions for each sample with 99% sequence identity (Quast et al., 2013).

### Analysis of diversity indices, data interpretation, and the PICRUSt analysis

Various diversity indices (Chao1, Shannon, Simpson, Pielou evenness, observed features, and Faith_pd) were calculated to analyse the bacterial diversity of each sample. The data have been interpreted for different outputs such as GraphPad Software, v. 6.0; San Diego, CA; Microsoft Excel; NCSS V20.0.2; and STAMP V2.1.3 (Parks et al., 2014). The functional prediction of the 16S rDNA genes was performed using the Phylogenetic Investigation of Communities by Reconstruction of Unobserved States (PICRUSt 2.0; version 2.4.1) algorithm (Douglas et al., 2020).

### Interpretation of the data and statistical analysis

The generated ASVs were analyzed for the overall bacterial composition in the gut microbiota of control and prenatally arsenic-treated animals at various taxon levels. The statistically significant differences at the level of tier I, tier II, and tier III were performed using a two-sided G-test (w/Yates)+Fisher's test at a 95% confidence interval to analyse the PICRUSt2 output using STAMP (https://stamp-software.com/). Data were depicted as mean ± SE normalized to control values in the figures. Comparative analyses among the three groups were performed using a one-way ANOVA (*p* < 0.05) using GraphPad software (GraphPad Software, v. 6.0; San Diego, CA) and Microsoft Excel.

## Results

### FastQC analysis and pre-processing of the reads

Amplicon libraries achieved from nine samples (three male samples from each group) revealed a total of 9,91,471 raw reads, which were converted to 5,12,543 high-quality non-chimeric reads using the DADA2 denoiser in the QIIME2 pipeline to generate amplicon sequence variants (ASVs) for taxonomy assignment (Supplementary Table 1).

### Taxonomic assignment

On taxonomic assignment, the filtered reads were assigned into nine phyla, 13 classes, 25 orders, 35 families, 53 genera, and 27 species.

### Dominant phyla

**Figure 1:**
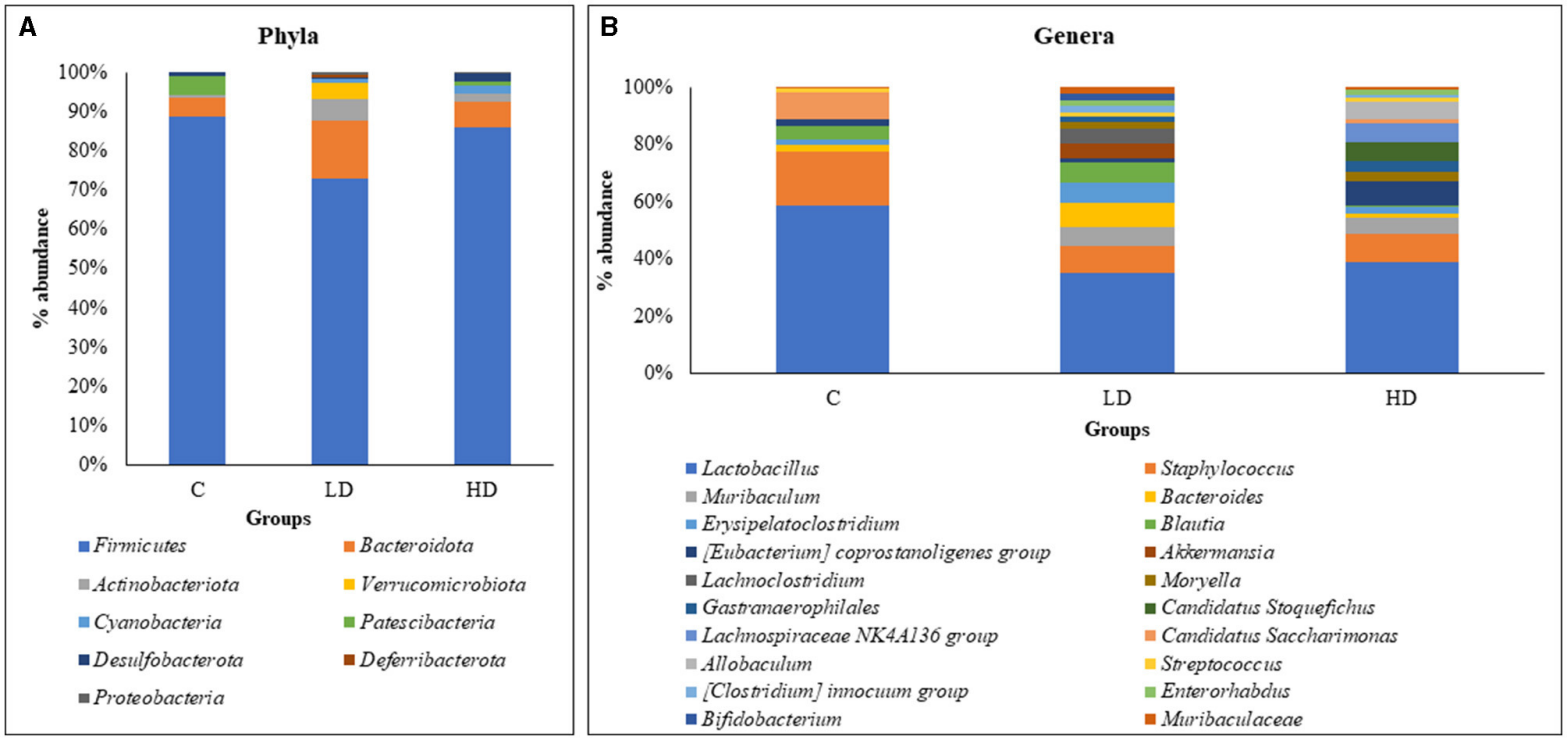
Relative percentage abundance at the level of phyla between C (control), LD (low dose), and HD (high dose) groups **(A)**. Relative percentage abundance of top 20 genera among the three groups (C, LD, and HD) **(B)**.

Overall data analysis revealed that *Firmicutes* (79.56%) were the most abundant phyla in the gut microbiota followed by *Bacteroidota* (10.57%), *Actinobacteriota* (5.58%), *Verrucomicrobiota* (2.14%), *Cyanobacteria* (1.29%), *Patescibacteria* (1.12%), and *Desulfobacterota* (1.01%). Other phyla were *Deferribacterota* (0.40%) and *Proteobacteria* (0.34%), which contributed <1%. Phyla *Verrucomicrobiota, Cyanobacteria, Deferribacterota*, and *Proteobacteria* did not appear in the control animals and were present only in either of the prenatally arsenic-treated groups (Figure 1A).

### Dominant genera

Of the 53 genera, *Lactobacillus* (33.87%) was the most abundant in the gut microbiota, followed by *Staphylococcus* (9.38%), *Muribaculum* (4.65%), *Bacteroides* (4.52%), *Erysipelatoclostridium* (4.4%), *Blautia* (4.28%), *Coprostanoligenes [Eubacterium]* (2.79%), *Akkermansia* (2.76%), *Lachnoclostridium* (2.36%), and *Moryella* (2.06%), which constituted the top 10 genera. Thirty-six genera were absent in the control group and observed in prenatally arsenic-treated groups only. Four genera (*Roseburia, Odoribacter, Prevotella*, and *RF39*) were detected only in the control group, while the remaining genera exhibited a portion below 2%, thus exhibiting low significance in modulating the physiology of the gut. The top 20 genus abundances have been shown in Figure 1B.

### Comparative analysis between control and arsenic-treated group

Out of the top nine phyla, *Firmicutes* (88.66%) and *Patescibacteria* (4.74%) showed relatively high dominance in the control group, i.e., without arsenic dose. Whereas in the LD group, *Bacteroidota* (14.78%), *Actinobacteriota* (5.27%), *Verrucomicrobiota* (4.02%), *Deferribacterota* (0.76%), and *Proteobacteria* (0.51%) were relatively dominant. In the HD group, *Cyanobacteria* (2.19%) and *Desulfobacterota* (2.28%) were relatively dominant.

Out of the 53 genera, *Lactobacillus* (52.77%), *Staphylococcus* (17.25%), *Candidatus Saccharimonas* (8.78%), *Alistipes* (3.41%), *Desulfovibrio* (0.82%), *Roseburia* (0.83%), *Odoribacter* (0.77%), *Prevotella* (0.43%), and *RF39* (0.27%) showed relatively high dominance in the control group. In the LD group, 29 genera showed relative dominance, out of which 15 were more than 1%. *Muribaculum* (5.61%), *Bacteroides* (6.87%), *Erysipelatoclostridium* (6.08%), *Blautia* (6.29%), *Akkermansia* (4.74%), *Lachnoclostridium* (4.05%), *Streptococcus* (1.41%), *[Clostridium] innocuum group* (2.05%), *Bifidobacterium* (2.19%), *Muribaculaceae* (1.75%), *Phascolarctobacterium* (1.96%), *[Ruminococcus] gnavus group* (1.86%), *Corynebacterium* (1.22%), *[Ruminococcus] torques group* (1.09%), and *Prevotellaceae UCG-001* (1.02%) were relatively dominant genera. Fourteen dominant genera had <1%. In the HD group, *[Eubacterium] coprostanoligenes group* (7.22%), *Moryella* (2.90%), *Gastranaerophilales* (2.97%), *Candidatus Stoquefichus* (5.63%), *Lachnospiraceae NK4A136 group* (5.60%), *Allobaculum* (5.20%), *Enterorhabdus* (1.93%), *Erysipelotrichaceae UCG-003* (2.72%), *Dubosiella* (3.07%), *Clostridia UCG-014* (1.60%), *Enterococcus* (1.45%), *Lawsonia* (2.44%), *Lactococcus* (1.27%), *Anaerostipes* (1.28%), and *Clostridium sensu stricto 1* (0.43%) were relatively dominant genera.

### Diversity indices

**Figure 2:**
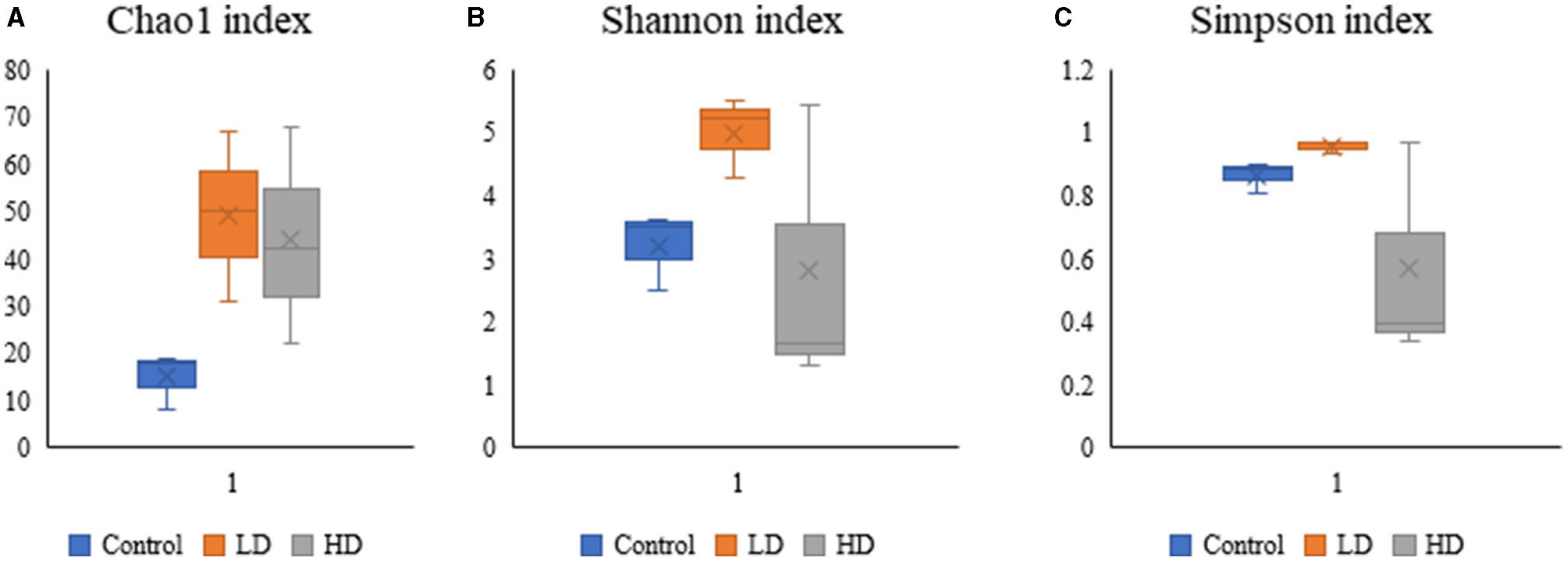
Box-plot analysis of the alpha diversity indices [Chao1 **(A)**, Shannon **(B)**, and Simpson **(C)**] among the three groups (C, LD, and HD).

Various diversity indices were analyzed for control and prenatally arsenic-exposed groups, i.e., LD and HD (Figure 2; Table 1). Prenatally arsenic-exposed groups LD and HD showed a higher richness of bacterial diversity than the control group. The highest average Chao1-index was observed in the LD group (49.33), followed by the HD (44.0.0) and the control groups (15). The highest average Shannon index was also observed in the LD group (5.01) as compared to the control (3.21) and the HD groups (2.81). Similarly, the average Simpson index was also maximum for the LD group (0.96) in comparison to the HD (0.86) and control groups (0.57). Average Pielou evenness was highest in the LD group (0.90), followed by the control (0.84) and the HD groups (0.50). Average observed features were also higher in the LD (48.67) and the HD (43.00) groups compared to the control group (15.00). Average Faith_pd was also measured, which was higher in the HD (8.37) and the LD (7.27) groups compared to the control (4.58) group.

**Table 1:** Various diversity indices and estimators obtained among control (C) and arsenic treated groups (LD and HD).

			**Average diversity indices and estimators**					
**Sample name**	**Dominant phyla (%)**	**Dominant genera (%)**	**Chao1**	**Shannon**	**Simpson**	**Pielou evenness**	**Observed features**	**Faith_pd**
Control (C)	*Firmicutes* (88.66%)	*Lactobacillus* (52.77%)	15.00	3.21	0.86	0.84	15.00	4.58
Low dose (LD)	*Firmicutes* (73.03%)	*Lactobacillus* (30.01%)	49.33	5.01	0.96	0.90	48.67	7.27
High dose (HD)	*Firmicutes* (85.81%)	*Lactobacillus* (32.58%)	44.00	2.81	0.57	0.50	43.00	8.37

### Shared bacterial phyla and genera among the groups

A total of nine bacterial phyla were identified among the three groups. Of them, only four phyla (*Firmicutes, Bacteroidota, Actinobacteriota*, and *Desulfobacterota*) were present in the gut microbiota of both the control and arsenic-exposed groups (LD and HD). At the genus level, 53 genera were identified among the three groups where *Lactobacillus, Staphylococcus, Bacteroides, Erysipelatoclostridium, Blautia, Coprostanoligenes group, Streptococcus, Muribaculaceae*, and *Desulfovibrio* were observed in the gut microbiota of both the control and the treated groups.

### Inter-individual differences among the groups

Inter-individual differences were observed at the phyla and genera levels among the control and arsenic-treated groups. At the phyla level, *Cyanobacteria* and *Proteobacteria* were seen in both the arsenic-treated groups, while their occurrence was missing in the control group. Moreover, *Verrucomicrobiota* and *Deferribacterota* were found only in the LD group. Whereas *Patescibacteria* was detected only in the control and HD groups. At the genera level, *Muribaculum, Moryella, Gastranaerophilales, [Clostridium] innocuum group, Enterorhabdus, Dubosiella, Clostridia UCG-014, Enterococcus, Prevotellaceae UCG-003, Gordonibacter*, and *Clostridium sensu stricto 1* were present in both the arsenic-treated groups but were not present in the control group. *Candidatus Stoquefichus, Lachnospiraceae NK4A136 group, Allobaculum, Lawsonia*, and *Anaerostipes* were the genera that were only present in the HD group.

### Functional analysis of the gut microbiota

The PICRUSt 2 (version 2.4.1) analysis was performed in QIIME2 on a command basis for exploring the bacterial functional role in the treated groups, and the control groups showed significant variations in the functionality. In tier I, the majority of the genes were involved in the metabolic pathways (50.05%), followed by the genes of genetic information processing (18.08%), environmental information processing (15.46%), cellular processes (7.67%), human diseases (6.02%), and organismal systems (2.73%; Supplementary Figure 1). Tier I analysis revealed that in a dose-dependent gene expression in all the functional groups, the HD group showed the highest value, followed by the LD group, and then the control group. Tier II analysis showed a significant abundance for the genes that belong to carbohydrate metabolism (23.08%), translation (49.23%), membrane transport (65.83%), cellular community prokaryotes (54.23%), endocrine system (41.54%), and antimicrobial drug resistance (31.15%; Supplementary Figures 2A–C, 3A–C). Twelve relevant pathways were chosen for further investigation. A comparative analysis of these genes showed that most pathways (10) were not altered due to arsenic treatment, either at lower or higher doses of arsenic. Only genes involved in non-alcoholic fatty liver diseases and insulin signaling pathways showed a decrease in their relative counts as compared to the control groups. In addition, neither of the genes was significantly different among the groups (*p* > 0.05).

## Discussion

It was demonstrated that an environmentally relevant level of arsenic exposure during the gestational period can perturb the normal community composition and functional pathways in the mouse gut microbiome. It has been previously described in chronic arsenic exposure studies that patterns of energy metabolism genes were altered (Lu et al., 2014; Chi et al., 2017), and genes involved in LPS synthesis (Chi et al., 2017; Yang et al., 2021), oxidative stress responses, and DNA repair were broadly increased due to long-term exposure to arsenic (Lu et al., 2014; Chi et al., 2017, 2018). In addition, arsenic exposure also enriched genes that encode conjugative transposon proteins, components of the multidrug efflux system, and the synthesis of multiple vitamins. As the exposure to low, environmentally relevant doses of arsenic during the gestation period culminates in the disruption of glucose homeostasis and energy metabolism-related genes, the current study explored whether it also affects the establishment and maintenance of the natural gut microbiome or not. The results of the study provide a new understanding of the effects of arsenic on the gut microbiome, especially at environmentally relevant doses. High-throughput 16S rRNA gene sequencing was used for profiling to study the impact of arsenic exposure on the gut microbiome and its metabolic profiles. In a study, 16S RNA-based analysis of the gut microbiome unveiled that perturbation in the gut microbiome enhances the arsenic bioaccumulation of its toxicity (Coryell et al., 2018). Our observations clearly show that arsenic exposure induced a noteworthy change in the gut microbiome composition of prenatally treated male mice, indicating that arsenic exposure not only disturbs gut bacteria at the abundance level but also substantially alters the metabolomic profile of the host, resulting in the disturbance of host metabolite homeostasis after arsenic exposure. Chi et al. (2017) observed that the 100-ppb arsenic dose for 13 weeks was sufficient to alter the bacterial diversity of the gut microbiome of mice. Therefore, the current findings may provide mechanistic insights regarding perturbations of the gut microbiome as a new mechanism of environmental chemical-induced human diseases. It may be hypothesized that these alterations might be due to the vertical transfer of arsenic-exposed gut microbiota from the mother to F1-generation after gestational arsenic exposure. In the F1-generation, which has acquired arsenic-exposed microbiota from its mother, along with other adaptations and epigenetic modifications, an increase in the diversity of gut microbiota was observed. In the normal gut microbiota, the relative abundance of both *Firmicutes* and *Bacteroidetes* was recorded, but a shift in the relative abundance of *Firmicutes* and *Bacteroidetes* was observed in obese vs. lean mice, with a statistically significant reduction in *Bacteroidetes* and an increase in *Firmicutes* in obese mice. Overall analysis revealed that *Firmicutes, Bacteroidota*, and *Actinobacteriota* constitute the major phyla as reported in several investigations on arsenic toxicity (Thursby and Juge, 2017; Binda et al., 2018; Magne et al., 2020) in the gut microbiome. The members of these phyla are prominently present in the gut microbiota of humans as well as mice. *Firmicutes* and *Bacteroidetes* exhibit a plethora of enzymes involved in carbohydrate metabolism (Turnbaugh et al., 2006). Moreover, the prevalence of *Lactobacillus, Staphylococcus, Muribaculum*, and *Bacteroides*-like genera further corroborates the findings of several investigations (Bervoets et al., 2013; Harakeh et al., 2016; Crovesy et al., 2017, 2020; Halawa et al., 2019; Tokarek et al., 2021; Zheng et al., 2022). Guo et al. reported an increase in *Firmicutes* and *Proteobacteria* and a decrease in *Bacteroidetes* after arsenic exposure, which is consistent with changes observed here in the two lowest doses (Richardson et al., 2018). In the current study, the highest dose also showed an increase in *Proteobacteria*, but not in *Firmicutes* or *Bacteroidetes* populations (Richardson et al., 2018).

The comparative analysis among the three groups (control, LD, and HD) showed the differences at the phyla level. *Firmicutes* were dominant in the control groups, which follows the routine pattern of the majority of the gut bacteriome (Chi et al., 2017). The depletion of *Firmicutes* in LD and HD groups indicates the effect of arsenic on the firmicute population and its alterations, which follows the findings of previous studies (Dheer et al., 2015; Wu et al., 2019). Lu et al. (2014) also reported a significant decrease in *Firmicutes* due to arsenic exposure, which plays a significant role in affecting energy harvesting pathways and short-chain fatty acid (SCFA) production. Several *Firmicutes* are known for butyrate production (Tremaroli and Bäckhed, 2012; Lu et al., 2014). SCFA such as propionate, butyrate, and acetate acts as the primary energy source for gut epithelial cells and promotes the first line of cellular defense (Vinolo et al., 2011). The abundance of *Bacteroidota* was prevalent in the LD group and exhibited a cluster of carbohydrate-utilizing enzymes such as *Firmicutes*. It may be due to the low dose of arsenic exposure to the LD group (0.04 mg/kg) as compared to the HD group (0.4 mg/kg). Dheer et al. (2015) observed a positive correlation between *Bacteroidetes* count and a higher dose of arsenic. In the HD group, a drastic depletion in *Firmicutes, Bacteroidetes*, and *Actinobacteria* was observed due to a higher dose of arsenic exposure. Studies examining alterations in the microbial composition after arsenic exposure have shown results that appear to conflict. However, Guo et al. found that providing mice with water containing arsenic increased the abundance of *Firmicutes* and decreased the abundance of *Bacteroidetes* (Richardson et al., 2018). *Proteobacteria* are often overrepresented in several intestinal and extraintestinal diseases, mostly with an inflammatory phenotype (Mukhopadhya et al., 2012; Rizzatti et al., 2017). At the late stages of life, the microbiota composition becomes less diverse and more dynamic, characterized by a higher *Bacteroides* to *Firmicutes* ratio and an increase in *Proteobacteria* (Biagi et al., 2010). Similar findings were observed at the genera level, where the control group harbored a higher count of the genera *Firmicutes* and *Bacteroidetes* in the control and LD groups. An increase in *Lactobacillus* in the control group was observed, whereas in the arsenic-exposed groups (LD and HD), the *Lactobacillus* count was significantly decreased. *Lactobacillus* spp. are known for their protective role in arsenic-induced health damage (Sanders et al., 2019; Du et al., 2020). *Lactobacillus* may acquire arsenic resistance by reverting the oxidative stress and the production of pro-inflammatory cytokines (de Matuoka et al., 2020). Du et al. (2020) observed the restoration of *Lactobacillus* spp. in the gut microbiome after 30 days of arsenic exposure in mice. *Lactobacillus* spp. exhibit antimicrobial and antioxidative probiotic activity to stabilize the gut microbiome (Sanders et al., 2019; Wu et al., 2019). The LD and HD groups showed several pathogenic bacteria, such as *Streptococcus, Prevotellaceae, Corynebacterium*, and *Enterococcus*, that altered bacterial diversity due to arsenic doses in mice. Besides, the overall diversity of the LD and HD groups showed variation compared to the control group. A long-term arsenic exposure of 6 months in adults reduced the count of *Muribaculum*; however, this genus was not observed in arsenic-treated groups only (Wang et al., 2020). Similarly, the count of *Dubosiella* significantly decreased after 30 days of arsenic exposure. Here, we observed the presence of *Dubosiella* in the treated group (Wang et al., 2020). The physiology of *Dubosiella* is not well-defined and needs to be further explored. *Enterococcus*, another unique genus of the treated group, belongs to the lactic acid bacteria (LAB) family, comprising both pathogenic and commensal microorganisms ubiquitous in the environment even as gut symbionts (Hanchi et al., 2018). Due to their tolerance to salts and acids, the strains of *Enterococcus* spp. are highly adapted to several food systems (Hanchi et al., 2018). Several arsenic-resistant strains of *Enterococcus* are known to assist in coping with high metal environments (Abrantes et al., 2011; Parsons et al., 2020).

In adult studies of metal exposures, a dose-dependent response in the alteration of the gut microbial community has been observed (Dheer et al., 2015; Gokulan et al., 2018). However, in the current investigation, a dose-dependent response in the alteration of gut microbiota was not detected. Whereas in metabolic pathways, there is an increase in pathways involved in carbohydrate metabolism, translation, membrane transport, cellular community prokaryotes, endocrine system, and antimicrobial drug resistance in a dose-dependent manner. This might be due to adaptation, epigenetic modifications, and transfer of altered microbial communities from directly arsenic-exposed mothers to the offspring during the gestation period, which needs to be further confirmed. The depletion of *Roseburia, Ruminococcaceae, Odoribacter*, and *Prevotella*-like genera in the control group identified them as arsenic-sensitive genera. *Prevotella* constitute the core bacteriome of the human gut (Nam et al., 2011; Bhute et al., 2016) and participate extensively in hydrolysing high-fiber-based polysaccharides. *Prevotella* spp. exhibit a repertoire of carbohydrate hydrolysing complexes (Yeoh et al., 2022). *Roseburia* is associated with the effects of colonic motility, immunity maintenance, and anti-inflammatory properties (Shao and Zhu, 2020). *Roseburia* spp. could also serve as biomarkers for symptomatic pathologies (e.g., gallstone formation) or as probiotics for the restoration of beneficial flora (Tamanai-Shacoori et al., 2017). The studies on *Prevotella* state that this genus was found to be enriched in the treated group. Whereas in the current investigation, it was present in the control group only (Wang et al., 2020).

In another study, Ashutosh et al. reported that *Prevotella* can suppress disease through the modulation of systemic immune responses (Mangalam et al., 2017). In addition, it was absent in the arsenic-treated group, which can play a role in metabolic deregulation. Whereas, *Odoribacter* showed a significant increase in the presence of cadmium and aluminum exposure (Zhai et al., 2017), which is contradictory to the current investigation, needs to be further examined, and may be attributed to the indirect effect of arsenic on prenatally treated mice.

The PICRUSt-based functional prediction showed the dominance of metabolic pathways (50.05%), which follows the pattern of several microbiome-based investigations. As it was a gut microbiome-based investigation, several genes of carbohydrate and fatty acid metabolism were assessed. It was observed that most pathways (10) were not altered due to arsenic treatment either at lower or higher doses of arsenic. Only genes involved in non-alcoholic fatty liver diseases and insulin-signaling pathways showed a decrease in their relative counts in the treated group as compared to the control groups. Frediani et al. (2018) reported a positive association between NAFLD and arsenic exposure. However, this investigation was not a microbiome-based study and therefore cannot be truly correlated with insulin-signaling pathways.

## Conclusion

Overall, we can conclude that only gestational arsenic exposure is sufficient to alter the gut microbial community in the progeny. The effects of prenatal exposure are manifested through an increased gene expression profile in the gut microbiota with most of the genes clustered in metabolism and its pathways. The outcomes of the study illustrate that arsenic exposure perturbs the gut microbiome composition and associated metabolic profiles in mice, which represents an early and crucial step in understanding how arsenic exposure affects the gut microbiome and its functions.

## Data availability statement

The data presented in the study are deposited in the NCBI repository, SRA accession number PRJNA929678.

## Ethics statement

The animal study was reviewed and approved by the Ethical Committee, CSIR-Indian Institute of Toxicology Research, Lucknow.

## Author contributions

SS and DCS conceived and planned the presented idea. SS, SG, AC, SB, and VS performed animal experiments and sequencing. SS, AS, DV, and DCS performed sequencing analysis and wrote the paper with input from all authors. DT supervised the statistical analysis. All authors listed have made a substantial, direct, and intellectual contribution to the work and approved it for publication.

## Supplementary material

Supplementary Figure 1 The PICRUSt analysis of tier I level shows the distribution of ASVs into various pathways according to the KEGG database.

Supplementary Figure 2 The PICRUSt analysis tier II shows the ASVs distribution in metabolic pathways **(A)**, genetic information processing **(B)**, and environmental information processing **(C)**.

Supplementary Figure 3 The PICRUSt analysis tier II shows the ASVs distribution in cellular processes **(A)**, human diseases **(B)**, and organismal systems **(C)**.
