# Supplementary material for: Analysis of gut bacteriome of in utero arsenic-exposed mice using 16S rRNA-based metagenomic approach
Source: Front Microbiol. 2023 Sep 29;14:1147505. doi: 10.3389/fmicb.2023.1147505 (PMC10570621; doi:10.3389/fmicb.2023.1147505)
Supplement: Supplementary Figure 1 — The PICRUSt analysis of tier I level shows the distribution of ASVs into various pathways according to the KEGG database. [file Data_Sheet_1.pdf]

Supplementary Table 1: Statistics of sequencing reads obtained from denoising using DADA2 in QIIME2

Input reads	Input reads	Filtered reads	Percentage of input passed filter reads	Denoised reads	Merged reads	Percentage of input merged reads	Non-chimeric reads	Percentage of input non-chimeric reads
**Control 1**	59912	33347	55.66	27550	17558	29.31	16610	27.72
**Control 2**	37275	21461	57.57	18804	11794	31.64	9757	26.18
**Control 3**	47343	28363	59.91	22508	13213	27.91	12536	26.48
**Low Dose 1**	204988	132051	64.42	119615	84617	41.28	58203	28.39
**Low Dose 2**	112379	71028	63.2	62473	44017	39.17	29805	26.52
**Low Dose 3**	87819	54354	61.89	46971	34719	39.53	21535	24.52
**High Dose 1**	233835	153450	65.62	141886	106916	45.72	57830	24.73
**High Dose 2**	83603	7262	8.69	6560	5940	7.11	5940	7.11
**High Dose 3**	124317	11227	9.03	10063	8861	7.13	8843	7.11

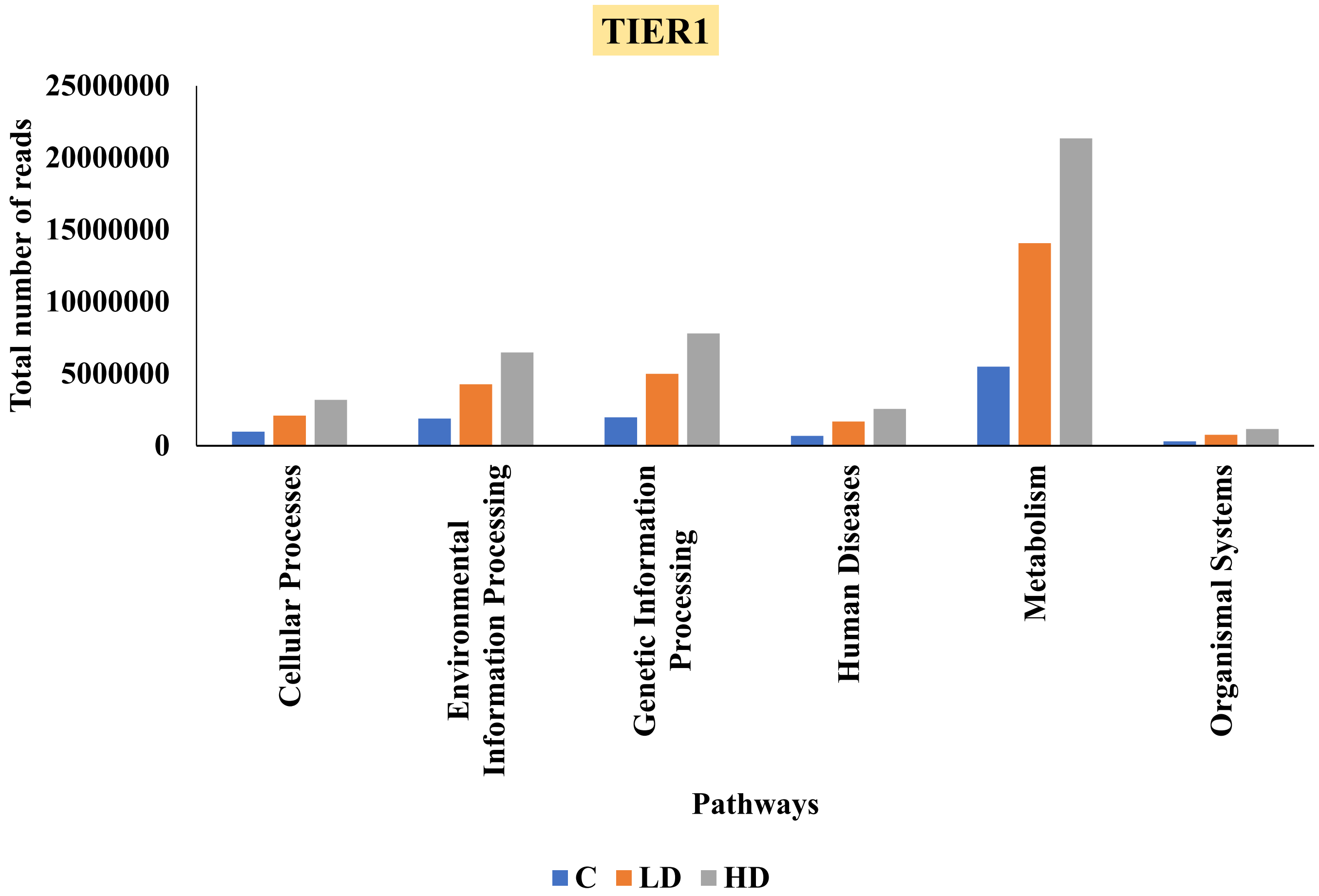

Supplementary Fig. 1

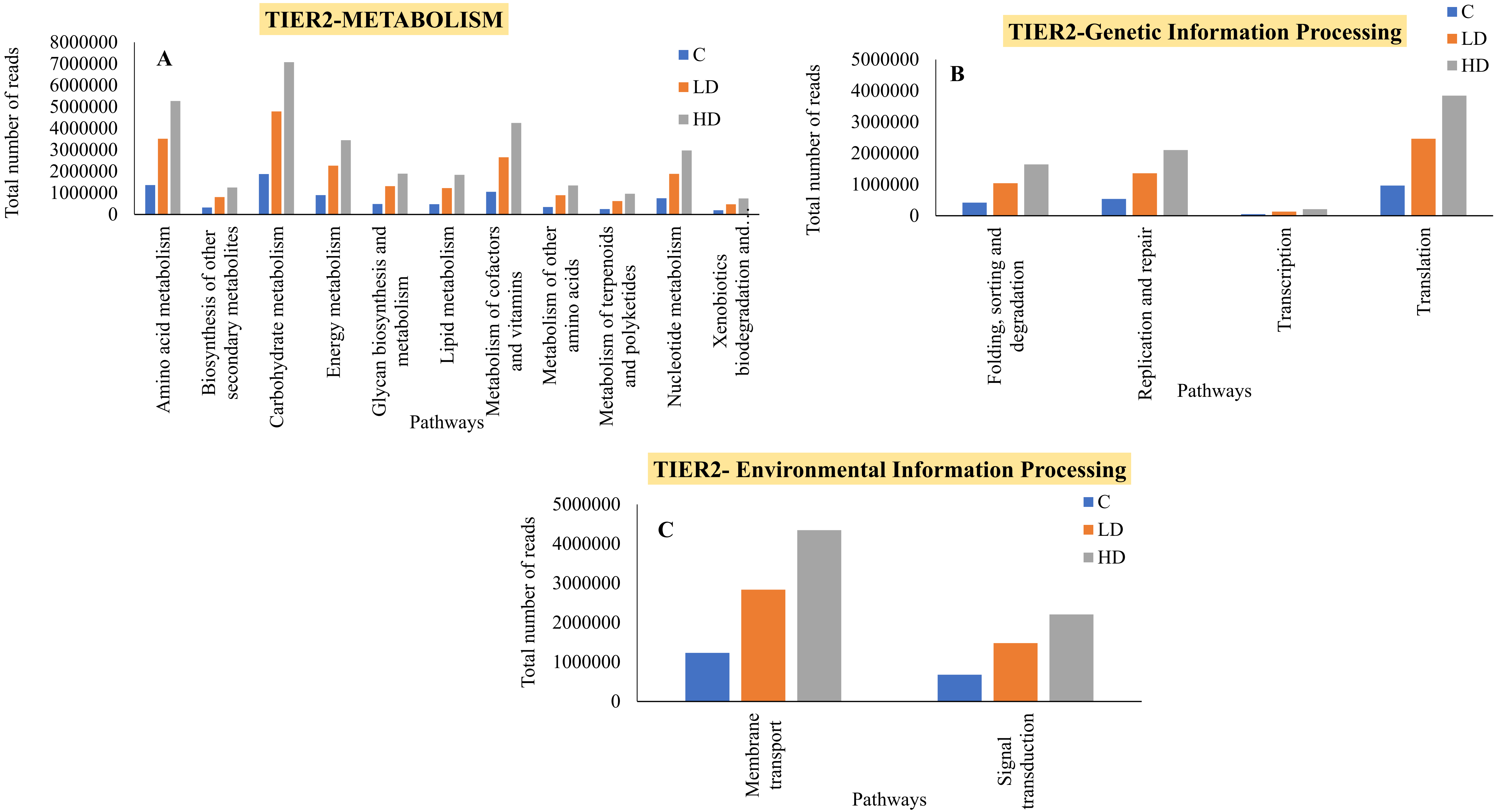

Supplementary Fig. 2

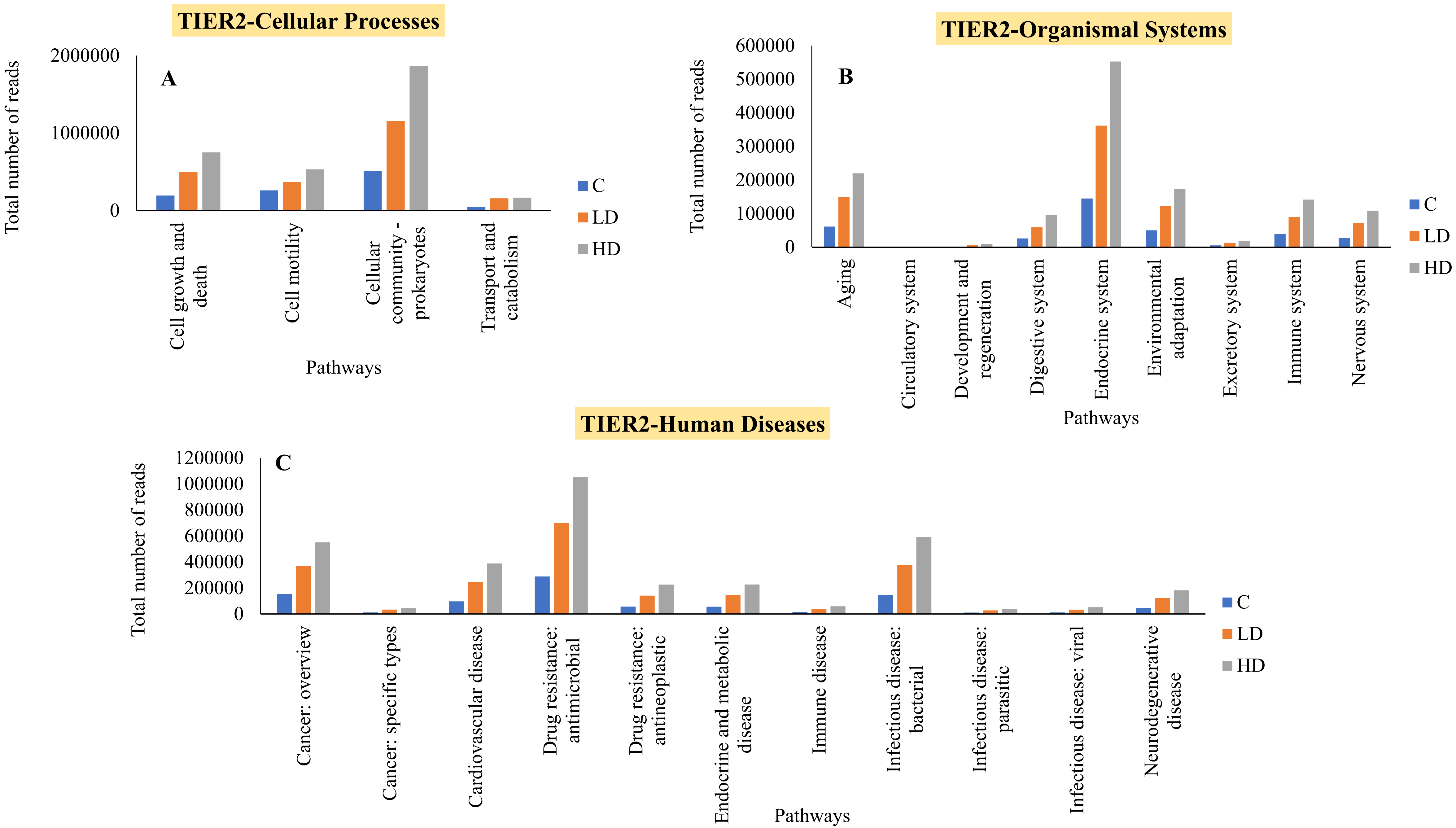

Supplementary Fig. 3
